# Bibliographical Notices

**Published:** 1839-01-26

**Authors:** 


### BIBLIOGRAPHICAL NOTICES.

*The Principles of Surgery, Volume Second: comprising the Surgical Anatomy of the Human Body, and its application to Injuries and Operations.* By JOHN BURNS, M. D., F. R. S., Regius Professor of Surgery in the University of Glasgow, &c. &c. London: 1838. pp. 585.

THE first volume of this work, published some years since, (1831,) was devoted to an exposition of the doctrines of inflammation, together with those actions and conditions which are commonly recognised by surgeons as its results, and contained some novel views of the author on this interesting question. In the present volume we have detailed, with much clearness and precision, the relative situations and especial connexions of those portions of the human body which may become the seat of operations, together with precise and judicious directions for performing them—or, in other words, the mechanical principles of surgery.

The high reputation of the Glasgow Professor will be increased by this publication.

---

*Statistical Report of the Sickness, Mortality, and Invaliding among the Troops in the West Indies, prepared from the Records of the Army Medical Department, and War Office Returns.* London: 1838.

This report comprises the medical statistics of the British army in the West Indies, during a period of twenty years. An immense mass of materials had in this time accumulated, the arrangement and analysis of which have lately been completed. The results are highly interesting, not only from their bearing upon military medicine and hygiene, but from the light they throw upon the medical history and topography of the West India islands. In the arrangement of the statistics, the entire group of islands has been divided into four sections, or commands:—the Windward and Leeward command; the Jamaica command; the Bahamas; and the Honduras command. We propose to take a rapid view of the comparative sickness and mortality in these different sections, dwelling more particularly upon certain points which are of special interest to the North American reader.

The Windward and Leeward command comprises the islands between 6° and 17° N. latitude, and 56° and 33° W. longitude. The annual mortality here is 93⅗ per 1000; in the same class of troops serving in Great Britain, during a period when the epidemics of cholera and influenza prevailed, the mortality did not rise above 17 per 1000, less than one-fifth of the former. The diseases which prevail most in this command, are—1. Fevers. 2. Disorders of the alimentary canal. 3. Abscesses and ulcers. 4. Wounds and Injuries. 5. Diseases of the lungs. 6. Diseases of the eyes. 7. Rheumatic disorders. 8. Venereal disorders. 9. Disorders of the brain; and, 10. Disorders of the liver. Fevers, therefore, are here the chief sources of sickness and mortality; and this mortality is chiefly occasioned by remittent and yellow fevers. In diseases of the stomach and bowels, the principal source of mortality is acute and chronic dysentery. In the acute stage, the mortality is moderate; but dysentery has a strong tendency to become chronic, when it is most unmanageable. With regard to diseases of the lungs, the statistics show, that, although they are more prevalent in Great Britain, they are more fatal in the West Indies. It is also proved, that *consumption* is more prevalent in this command than in Great Britain,—attacking 12 per 1000 annually in the Windward and Leeward islands, and only 5½ per 1000 in Great Britain. The important inference from these data is, that the influence of hot climates upon consumption is hurtful; and that to send a consumptive patient to them, is to hasten the progress of his disorder! On the subject of diseases of the brain, it is interesting to know that the great mortality is from the brain-fever of drunkards,—15 out of every 1000 *white* troops suffering from it, and 2 out of 16 dying. Among the native black troops, the prevalence of this fever was only at the rate of 13 per 10,000, of whom there died 1 in 6⅓. From the returns, it appears that the coloured troops enjoy no immunity from other cerebral and meningeal disorders; and we are compelled to ascribe their remarkable exemption in this particular to their superior temperance. As regards diseases of the liver, it is worth noting, that they are by no means so prevalent here as in the East Indies.

In the Jamaica command, the annual average mortality is 143 per 1000, greater than in the Windward and Leeward command; although the actual amount of sickness is less, in the ratio of 18 to 19. During the period in question, there were four severe epidemics of yellow fever, to which much of the mortality is due: fevers are, indeed, the main cause of the great mortality in Jamaica. Next in order of prevalence, but not of fatality, are disorders of the alimentary canal. Diseases of the lungs are less frequent, but more fatal than the last mentioned. The facts developed from the statistics of the Jamaica command, establish the greater frequency aud fatality of pulmonary consumption in Jamaica than in Great Britain; and these further show, that *double* the number of consumptive cases originate in the former climate, though catarrhal affections are comparatively rare. The conclusion is, of course, most unfavourable to the practice of sending phthisical patients to Jamaica. Upon diseases of the brain, intemperance exercises the same baneful influence in Jamaica, as in the first mentioned command: one-fourth of the whole are the direct effects of the abuse of spirituous liquors. The blacks enjoy here, also, a singular immunity from the brain-fever of drunkards.

On the Bahama and Honduras stations, the general character of the results is similar to those above deduced, and confirm points there alluded to. From the tables of the entire group, we derive the important and not generally received conclusions—first, that the mortality in the West Indies is greatest among the young, and decreases with the advance of life; secondly, that the health deteriorates in proportion to the length of residence, and that what is termed acclimatization, far from affording security, increases the risk of life.

The general deductions of the gentlemen who have had charge of the arrangements of these statistics, Mr. Marshall and Captain Tulloch, are entitled to an extended notice, and we are convinced that we shall not misapply our space, by giving them at length. For the data of our brief analysis, we are indebted to the Edinburgh Medical and Surgical Journal.

"It has been supposed by many that the diseases which prove so fatal to Europeans in these latitudes, especially fevers, are, if not a necessary, at least a very general consequence of continued exposure to a high temperature. The sufficiency of this, however, as a uniform cause of sickness and mortality, is contradicted by the fact, that these vary considerably in different stations, the mean temperature of which is nearly alike. The range of the thermometer, for instance, in Antigua and Barbadoes, is rather higher than in Dominica, Tobago, Jamaica, or the Bahamas; yet we find that the troops in the latter stations suffer nearly three times as much as those in the former. The preceding pages also afford several instances in which epidemic fever made its appearance, and raged with the utmost virulence during the winter months—a circumstance not likely to have taken place if that disease had originated in increased temperature. We may also state that the epidemic fevers which prevailed at Grenada in 1793, and at St. Christopher's in 1812, two of the most fatal which ever appeared in the West Indies, commenced, the former in March, and the latter in February, and continued with unabated violence during the whole of the cold season.

"If elevated temperature was an essential cause of the mortality to which Europeans are liable in this climate, we might expect it in every year to produce similar effects; whereas, on the contrary, it appears, from the tabular statements in the preceding Report, that the mortality in one year is sometimes twenty times as high as in another, without any perceptible difference in the range of temperature. This fact has already attracted the notice of some medical authors, who in treating of yellow fever, adduce instances of various epidemics both within and beyond the tropics, during which the temperature was not above the average, and was sometimes even a little below it, and inversely where the existence of a high temperature was not attended with the prevalence of fever.*

"In accounting for the unhealthiness of these colonies, great influence has been ascribed to excess of moisture, and the inference derives plausibility from various facts in the history of tropical fevers, especially their great prevalence along the sea coast, at the outlets of rivers, and in the vicinity of swampy level grounds. This hypothesis, however, seems at variance with the facts contained in the previous Report; for if the mortality of the troops depended materially on the influence of moisture, we might expect it to attain its maximum in those stations where the fall of rain was the greatest, whereas the average mortality of the troops in Jamaica is at least double that which prevails among those in British Guiana, though the quantity of rain which falls in that island is little more than half as great; and in the preceding pages there are adduced many instances in which epidemic fever has broken out, and raged with great violence, at a period when no rain had fallen for several months; nay, in some stations, a dry, in others a wet season, is looked on as the most unhealthy—an anomaly not likely to occur, if excess of moisture was uniformly an essential cause of insalubrity.

"It must also be remembered that this excess of moisture is not confined to the West Indies, but is a general characteristic of all tropical regions: and were it is so productive of disease in the Western hemisphere, the same effect might be expected to ensue from it in the East; whereas, on the contrary, the Malabar Coast, which is deluged by rain for six months in the year, is generally one of the most healthy quarters in the Madras Presidency.

"That neither heat nor moisture can be the primary causes which influence the health of troops in the West Indies, is at once established by referring to the comparative view of the ratio of mortality in each year at every station, exhibited in Tables XXXIV. and LX., in which there are numerous instances of two adjacent islands, or even of two contiguous stations in the same island, being subject in an equal degree to the operation of these agencies, and yet while the one has been desolated by the ravages of fever, the other has been enjoying a degree of salubrity equal to that of Great Britain.

"Though heat and moisture are not the primary causes of fever, however, it is highly probable their operation tends, in some measure, to increase its intensity. The tables, illustrating the influence of the seasons on the health of the troops in each station, show that the greatest number of admissions into hospital, and deaths, has, on the average of a series of years, (though not uniformly or equally in each year,) taken place in those months when the greatest degree of heat was combined with the greatest moisture; and it may be observed as a striking exemplification of this fact, that as the sun proceeds northward in the ecliptic, carrying heat and moisture in his train, the period generally termed the unhealthy season, is later in the northern colonies than in those to the south.

"The unhealthy character of that period of the year in which the greatest degree of heat and moisture is combined, is not, however, confined to the West Indies, but extends also to the East, as well as over a large portion of the Northern temperate zone. In the Mediterranean stations, particularly, the admissions into hospital and deaths among the troops, average nearly twice as high between July and October, as during any other months of the year. Even in Canada, the same peculiarity is observable, though not in so marked a degree; and conversely in stations southward of the equator, that period of the year, which on the north of the line is the most unhealthy, becomes in the south the most salubrious, in consequence of the seasons being reversed. The calculations on which these statements are founded, will be submitted in subsequent Reports. At present we merely advert to them in order to guard against the error of referring to the climate of the West Indies exclusively, phenomena which are common not only to other tropical regions, but also to those of the temperate zone.

"A knowledge of this fact at once overturns a plausible hypothesis, which attributes the un-

* Craigie, Practice of Physic, pp. 224, 226, 227.

healthy character of the West Indies during what is termed the sickly season, viz., from July to October, to the want of the free ventilation afforded by the trade-winds during the rest of the year, but which at this period either cease altogether, or become very irregular. But though these two events, the failure of the trade-winds and the increase of sickness and mortality, take place at corresponding periods, the latter can never be regarded as a necessary consequence of the former, when we find that in other quarters of the globe, beyond the range of the trade-winds, that is, in countries north of the 30th and 32d degrees N. L., and in which ventilation is quite as perfect at that period as at any other, the unhealthy nature of these months is marked as strongly as in the West Indies.

"This same fact strikes also at the root of another hypothesis, which attributes the sickly season in these regions to some morbific principle generated in the vast forests and savannahs of the South American continent, and wafted to these islands by the south-westerly winds which generally prevail during that period. Besides, were this hypothesis correct, we might expect that British Guiana would, from its proximity to this cause of disease, be most subject to its operation, and consequently the most unhealthy; and that the colonies further to the north, being least exposed to it, would enjoy the greatest degree of salubrity. The result of our investigations into the comparative mortality in each colony shows, however, that their relative salubrity is by no means affected by their proximity to, or distance from that continent.

"Some who are conscious of the difficulty of accounting for the unhealthy character of these colonies by the operation of general causes, endeavour to trace it to the influence of local circumstances, in particular to exhalations or emanations from the soil. To illustrate, therefore, the nature and extent of the operation of this alleged cause, we have stated, as accurately as our information will admit, the physical and geological characters of the soil in each island, and in the immediate vicinity of each station; and by comparing these with the mortality there, have ascertained that at many stations, where the soil appears exactly the same, the rate of mortality is very different; and at others, where the soilis very different, the rate of mortality is much the same. It is also to be observed that, while the soil and its physical characters are the same in every year, the sickness and mortality are extremely variable, and only in certain seasons and years attain to an extraordinary degree of intensity. It frequently happens, too, that a station which has been remarkable for its sickly character for one or two seasons, becomes, without any perceptible reason, just as remarkable for i salubrity, which could scarcely happen if the cause of that sickness and mortality existed in the soil, which was constantly there to produce it.

"The agency, real or supposed, of marshes, is liable to a similar objection. That the vicinity of marshes, swamps, and lagoons, is generally subject to fevers, both of the intermittent and the remittent type, is a fact sufficiently established by multiplied experience, both in tropical countries and within the temperate zones. But that remittent or yellow fever may be generated where no such cause is in operation to produce it, and that consequently it is impossible to establish a necessary connexion between the cause and the appearance of that disease, is sufficiently established by the fact that the sickness and mortality in British Guiana and Honduras, where swamps and marshes most abound, are considerably less than at Up-Park Camp, and several of the other stations in Jamaica, remote from the operation of any such agencies.

"The same remark may be applied to excessive or rank vegetation, to the influence of which much of the sickness and mortality at some of the stations has been ascribed. To both of these causes, indeed, the remark already made regarding the influence of the soil, is strictly applicable. The marshy lands and the rank vegetation exist at many of the stations in every year, whereas the disease, which is represented to proceed from them, is only of occasional occurrence, and the foregoing Report shows that in some years the extent of mortality has been ten times as great as at others, when the degree of heat and moisture by which the marshy soils and vegetation are most likely to have been affected, have been much the same."

